# Angioleiomioma oral: relato de um caso e revisão dos achados atuais

**DOI:** 10.1590/1677-5449.000417

**Published:** 2017

**Authors:** Luiz Arthur Barbosa da Silva, Ana Miryam Costa de Medeiros, Patrícia Teixeira de Oliveira, Éricka Janine Dantas da Silveira, Márcia Cristina da Costa Miguel

**Affiliations:** 1 Universidade Federal do Rio Grande do Norte – UFRN, Programa de Pós-graduação em Patologia Oral, Natal, RN, Brasil.

**Keywords:** angioleiomioma, diagnóstico, imuno-histoquímica

## Abstract

O angioleiomioma é uma neoplasia benigna que, a partir da nova classificação da OMS (2013) para os tumores de tecidos moles, deixou de ser considerado um tumor de origem muscular lisa, passando a ser considerado um tumor de origem perivascular. Raramente os angioleiomiomas ocorrem na cavidade oral. A lesão é tratada cirurgicamente, com prognóstico considerado favorável. Este trabalho revisa os casos de angioleiomioma oral relatados na literatura nos últimos 5 anos e descreve esse tumor em um homem de 44 anos que apresentou um nódulo assintomático localizado em lábio superior, com evolução de 6 meses. As hipóteses diagnósticas foram de adenoma pleomórfico e adenoma canalicular. A lesão foi submetida à biópsia e análise histopatológica e imuno-histoquímica (S100, CD34, α-SMA, H-caldesmon e desmina) confirmaram o diagnóstico de angioleiomioma. Destacamos a imuno-histoquímica como um importante método auxiliar no diagnóstico diferencial do angioleiomioma com outras lesões e, principalmente, com o miopericitoma.

## INTRODUÇÃO

O angioleiomioma é uma neoplasia benigna que, a partir da mais recente classificação da Organização Mundial de Saúde (OMS) (2013) para os tumores de tecido mole, deixou de ser considerado um tumor de origem muscular lisa, passando a ser considerado um tumor de origem perivascular^1^. A etiologia dessa lesão permanece incerta, mas hipóteses sobre a participação de pequenos traumas, estase venosa, disfunções hormonais e alterações genéticas tem sido postuladas^2^^,^ 3.

A cavidade oral raramente é acometida por angioleiomiomas, sendo o lábio o sítio mais frequente, seguido pelo palato, mucosa jugal e língua^4^^-^ 6. Geralmente, os pacientes diagnosticados são adultos de meia-idade, com predileção por indivíduos do sexo masculino^4^^,^ 7 ^,^ 8.

Clinicamente, o angioleiomioma oral é caracterizado como um nódulo submucoso, de consistência firme, com crescimento lento e, na maioria dos casos, apresentando tamanho de até 2 cm de diâmetro^8^^,^ 9.

Microscopicamente, apresenta-se como uma lesão bem circunscrita, apresentando espaços vasculares de tamanhos e formas distintos, além de células musculares lisas com morfologia variada e dispostas em feixes desorganizados intercalados por fibras colágenas^10^. O angioleiomioma faz diagnóstico diferencial com o miofibroma, o neurofibroma, o neurilemoma, o leiomiossarcoma e, principalmente, com o miopericitoma. Os achados morfológicos, somados aos imuno-histoquímicos, são úteis para diferenciá-los^5^^,^ 11 ^,^ 12. O tratamento mais indicado consiste na excisão cirúrgica conservadora^6^^,^ 13.

O objetivo deste estudo foi descrever os achados clínicos, morfológicos e imuno-histoquímicos de um caso de angioleiomioma oral e comparar tais achados com os relatos de casos publicados nos últimos 5 anos na literatura científica especializada utilizando a base de dados PubMed.

## DESCRIÇÃO DO CASO

**Figura 1:**
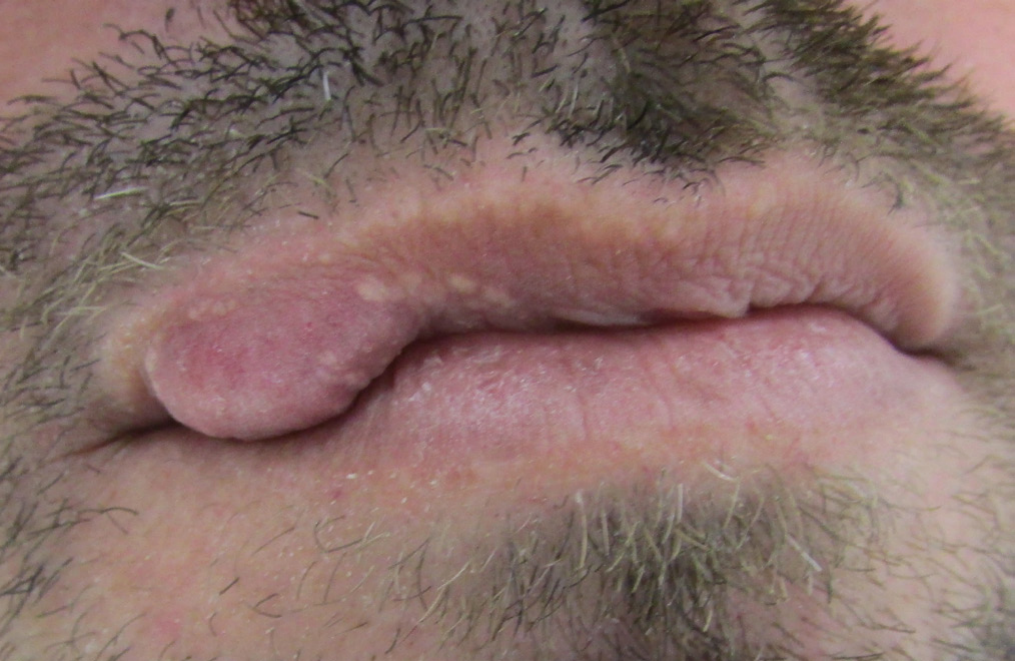
Aspecto clínico: lesão nodular em lábio superior.

**Figura 2:**
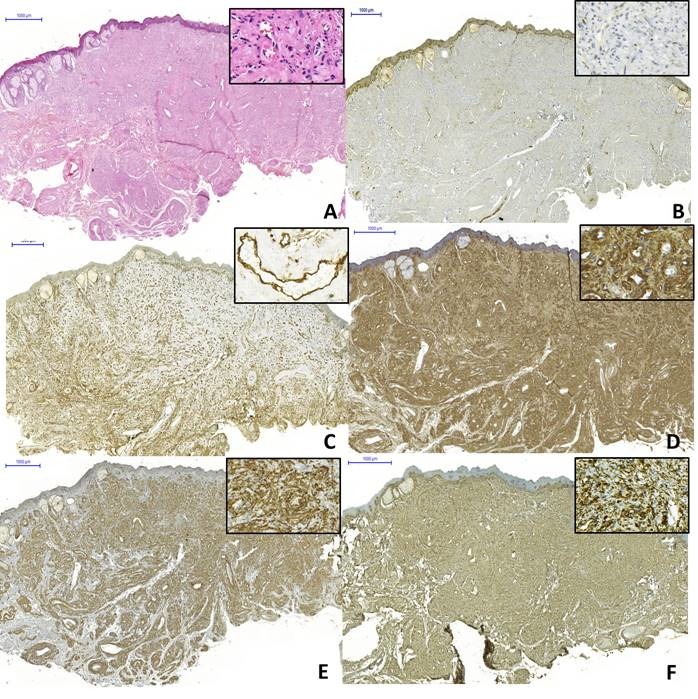
Aspectos morfológicos e imuno-histoquímicos do angioleiomioma. (A) proliferação de células com morfologias variadas (ovoides, fusiformes e onduladas) dispostas em feixes desorganizados e circundando múltiplos vasos sanguíneos (Hematoxilina & eosina, 100 µm); (B) Ausência de imunomarcação das células tumorais para proteína S100; (C) Positividade para CD34 restrita à parede vascular; (D-F) Imunomarcações fortes e difusas, respectivamente, para as proteínas α-SMA, H-caldesmon e desmina (100 µm).

Paciente do sexo masculino, 44 anos de idade, melanoderma, procurou um serviço de diagnóstico oral apresentando um aumento de volume assintomático, de superfície lobulada, normocrômico, com consistência fibrosa, localizado no lado direito do lábio superior, com evolução de aproximadamente 6 meses (Figura 1). Suas histórias médica e familiar não foram relevantes. Após exame clínico, foram levantadas as hipóteses diagnósticas de adenoma canalicular e adenoma pleomórfico. Sob anestesia local, a lesão foi submetida à biópsia excisional sem intercorrências. O espécime removido foi fixado em formol a 10% e enviado ao Laboratório de Anatomia Patológica. Os cortes histológicos revelaram uma neoplasia benigna de origem mesenquimal, localizada em região subepitelial, caracterizada pela proliferação de células com morfologia variada (ovoides, fusiformes e onduladas), dispostas em feixes desorganizados que circundavam múltiplos vasos sanguíneos de calibres distintos (Figura 2A). As hipóteses clínicas de neoplasias benignas de glândula salivar (adenoma pleomórfico e adenoma canalicular) foram descartadas. Para confirmação diagnóstica, foi necessária a realização de reações imuno-histoquímicas (Tabela 1). As células tumorais não evidenciaram nenhuma marcação para S100 (Figura 2B) e a positividade de CD34 mostrou-se restrita à parede de vasos sanguíneos (Figura 2C). As células tumorais mostraram marcação intensa para α-SMA (Figura 2D), além de imunopositividade para H-caldesmon (Figura 2E) e desmina (Figura 2F). A partir de tais achados foi estabelecido o diagnóstico de angioleiomioma oral. O paciente encontra-se há 15 meses em acompanhamento clínico sem sinais de recidiva da lesão.

**Tabela 1:** Painel imuno-histoquímico.

Anticorpo	Fabricante	Diluição e incubação	Padrão
S100	Dako	1:2000	Negativo para células tumorais
		60 minutos	
CD34	Dako	1:200	Restrito à parede de vasos
		60 minutos	
α-SMA	Dako	1:300	Forte e difuso
		Overnight	
H-caldesmon	Dako	1:400	Forte e difuso
		60 minutos	
Desmina	Dako	1:400	Forte e difuso
		60 minutos	

## DISCUSSÃO

De acordo com a OMS, os angioleiomiomas são tumores benignos dérmicos ou subcutâneos compostos de células musculares lisas bem diferenciadas que se organizam ao redor de diversas estruturas vasculares^14^. Sua etiologia ainda permanece incerta. Apesar de os estudos genéticos relacionados à origem das lesões perivasculares ainda serem limitados, mutações dos genes BRAF, NF1, NOTCH2 e NOTCH3 estão sendo investigadas^1^. Além disso, a expressão do receptor de estrogênio e do receptor de progesterona tem sido avaliada em angioleiomiomas, diante da possibilidade da participação de alterações hormonais como fator etiológico^2^.

Os angioleiomiomas podem ocorrer em qualquer área do corpo, porém a maioria dos casos acomete as extremidades, principalmente membros inferiores, seguidas por cabeça e tronco^8^. Também são relatadas altas incidências no útero, trato gastrointestinal e pele^4^^,^ 6. Os angioleiomiomas são raros na cavidade oral, diante da escassez de músculo liso nessa região^9^. Esses tumores parecem surgir a partir da camada média de pequenos vasos, de anastomoses arteriovenosas ou de células musculares lisas das papilas circunvaladas da língua^5^^,^ 12. Apesar de o lábio ser relatado como o sítio da cavidade oral mais comumente acometido por angioleiomiomas^4^^-^ 6, de acordo com os relatos da literatura especializada nos últimos 5 anos e apresentados na Tabela 2, a gengiva foi o sítio anatômico mais afetado, representado 29,4% dos casos.

**Tabela 2:** Casos de angioleiomiomas orais relatados nos últimos 5 anos na literatura (2011-2016).

Autores	N	Gênero	Idade (Anos)	Localização anatômica	Sintomatologia
Presente estudo (2016)	1	Masculino	44	Lábio inferior	Assintomático
Arpağ et al.^5^	2	Masculino	25	Gengiva	Assintomático
		Feminino	55	Gengiva	
Bajpai et al.^13^	1	Masculino	39	Gengiva	Assintomático
Ishikawa et al.^6^	1	Masculino	51	Língua	Assintomático
Inaba et al.^2^	1	Feminino	45	Mucosa jugal	Assintomático
Osano et al.^3^	1	Masculino	45	Mucosa jugal	Assintomático
Ranjan e Singh^9^	1	Feminino	45	Gengiva	Assintomático
Tsuji et al.^4^	1	Masculino	79	Palato duro	Assintomático
Eley et al.^8^	1	Masculino	39	Palato duro	Assintomático
Menditti et al.^15^	1	Masculino	14	Gengiva	Assintomático
Gueiros et al.^10^	3	Masculino	53	Lábio superior	Assintomático
		Masculino	54	Lábio inferior	Assintomático
		Masculino	66	Lábio superior	Assintomático
Mahima et al.^12^	1	Masculino	57	Região retromolar	Assintomático
Reddy et al.^7^	1	Masculino	9	Mandíbula	Assintomático
Vidaković et al.^16^	1	Feminino	85	Parótida	Assintomático

N: número de casos.

O angioleiomioma oral parece ser mais comum em pacientes do sexo masculino^4^^,^ 8, com uma relação homem:mulher de 3:1^3^. Normalmente, o pico de incidência do tumor está entre a 4ª e a 6ª décadas de vida^8^^,^ 10; por outro lado, já existem relatos de casos em pacientes pediátricos^7^. No levantamento apresentado na Tabela 2, observa-se que 76,4% dos tumores ocorreram em homens, com a faixa etária dos pacientes variando de 9 a 85 anos e média de idade de 47,3 anos. No presente caso, o paciente era do sexo masculino e tinha 44 anos de idade.

Os achados clínicos do caso por nós relatado corroboram os previamente descritos na literatura. Os angioleiomiomas apresentam-se como nódulos submucosos de crescimento lento e assintomático, bem delimitados, móveis, ocasionalmente com coloração azulada, de superfície íntegra, medindo, geralmente, 2 cm de diâmetro^7^^,^ 9.

O perfil imuno-histoquímico aqui apresentado confirma a origem muscular das células neoplásicas desse tumor, uma vez que foram notadas imunomarcações fortes e difusas para proteínas miogênicas, como α-SMA, H-caldesmon e desmina. O principal diagnóstico diferencial do angioleiomioma deve ser feito com o miopericitoma, uma vez que essas lesões apresentam achados histopatológicos sobrepostos^9^. Em um estudo avaliando 122 casos de angioleiomiomas e 12 casos de miopericitoma, na tentativa de determinar o perfil imuno-histoquímico característico desses dois tumores, Matsuyama, Hisaoka e Hashimoto^11^ observaram que α-SMA, HHF-35 e H-caldesmon apresentaram o mesmo padrão de imunomarcação em ambas as lesões, enquanto a desmina mostrou-se negativa em 75% dos casos de miopericitoma e em apenas 17,1% dos casos de angioleiomioma. Portanto, a desmina parece ser um marcador útil na diferenciação entre essas duas lesões. Nessa mesma perspectiva, do ponto de vista imuno-histoquímico, a desmina também pode ser útil para diferenciar angioleiomiomas de miofibromas, uma vez que em miofibromas as células neoplásicas são negativas para esse marcador^9^.

O padrão de imunomarcação da proteína S100 também é útil para distinguir o diagnóstico do angioleiomioma de neoplasias como o neurofibroma e o neurilemoma, visto que, além do padrão morfológico dessas lesões, a falta de reação dessa proteína para as células tumorais no angioleiomioma é totalmente divergente com o que é encontrado em lesões de origem neural^15^.

A diferenciação histopatológica entre o angioleiomioma e o leiomiossarcoma de baixo grau pode ser difícil. Dentre outros parâmetros, os leiomiossarcomas podem ser identificados por apresentarem uma contagem de 5 a 10 mitoses por campo, núcleos celulares com terminação romba e focos de necrose. Assim, um cuidadoso acompanhamento dos pacientes a longo prazo deve ser realizado diante da dúvida do diagnóstico entre essas duas entidades^3^^,^ 15.

O tratamento de escolha para o angioleiomioma oral é a excisão cirúrgica conservadora. Raras recidivas foram descritas, provavelmente em decorrência da excisão cirúrgica incompleta da lesão. Não existem relatos de transformação maligna e o prognóstico dos pacientes é considerado excelente^6^^,^ 16. O presente paciente encontra-se há 15 meses sem evidências de recidiva da lesão.

Salienta-se a importância do conhecimento da reclassificação do angioleiomioma como tumor de origem perivascular, segundo a OMS^14^, sendo necessários mais estudos genéticos para melhor esclarecer sua real etiologia. Destaca-se a imuno-histoquímica como um importante método auxiliar no correto diagnóstico do angioleiomioma, principalmente no que diz respeito ao diagnóstico diferencial com outras lesões, de maneira especial com o miopericitoma.

## References

[1] Fletcher CD (2014). The evolving classification of soft tissue tumours - an update based on the new 2013 WHO classification. Histopathology.

[2] Inaba T, Adachi M, Yagisita H (2015). A case of angioleiomyoma in the buccal space. Odontology.

[3] Osano H, Ioka Y, Okamoto R (2015). Angioleiomyoma of the cheek: a case report. J Oral Sci.

[4] Tsuji T, Satoh K, Nakano H, Kogo M (2014). Clinical characteristics of angioleiomyoma of the hard palate: report of a case and an analysis of the reported cases. J Oral Maxillofac Surg.

[5] Arpağ OF, Damlar I, Kılıç S, Altan A, Taş ZA, Özgür T (2016). Angioleiomyoma of the gingiva: a report of two cases. J Korean Assoc Oral Maxillofac Surg.

[6] Ishikawa S, Fuyama S, Kobayashi T, Taira Y, Sugano A, Iino M (2016). Angioleiomyoma of the tongue: a case report and review of the literature. Odontology.

[7] Reddy B, Rani BS, Anuradha C, Chandrasekhar P, Shamala R, Lingamaneni K (2011). Leiomyoma of the mandible in a child. J Oral Maxillofac Pathol.

[8] Eley KA, Alroyayamina S, Golding SJ, Tiam RN, Watt-Smith SR (2012). Angioleiomyoma of the hard palate: report of a case and review of the literature and magnetic resonance imaging findings of this rare entity. Oral Surg Oral Med Oral Pathol Oral Radiol.

[9] Ranjan S, Singh KT (2014). Gingival angioleiomyoma-infrequent lesion of oral cavity at a rare site. J Oral Maxillofac Pathol.

[10] Gueiros LA, Romañach MJ, Pires-Soubhia AM, Pires FR, Paes-de-Almeida O, Vargas PA (2011). Angioleiomyoma affecting the lips: report of 3 cases and review of the literature. Med Oral Patol Oral Cir Bucal.

[11] Matsuyama A, Hisaoka M, Hashimoto H (2007). Angioleiomyoma: a clinicopathologic and immunohistochemical reappraisal with special reference to the correlation with myopericytoma. Hum Pathol.

[12] Patil K, Mahima VG, Srikanth HS (2011). Recurrent oral angioleiomyoma. Contemp Clin Dent.

[13] Bajpai M, Pardhe N, Kumar M (2016). Angioleiomyoma of gingiva masquerading as pyogenic granuloma. J Coll Physicians Surg Pak.

[14] Hisaoka M, Quade B, Fletcher CD, Bridge JA, Hagendoorn PCW, Martens F (2013). Angioleiomyoma. WHO classification of tumor of soft tissue and bone.

[15] Menditti D, Laino L, Nastri L, Caruso U, Fiore P, Baldi A (2012). Oral angioleiomyoma: a rare pathological entity. In Vivo.

[16] Vidaković B, Knezević AK, Manojlović S, Knezević G (2011). Angiomyoma of the cheek. Coll Antropol.

