# Supplementary material for: Angioleiomioma oral: relato de um caso e revisão dos achados atuais
Source: J Vasc Bras. 2017 Jan-Mar;16(1):68–72. [Article in Portuguese] doi: 10.1590/1677-5449.000417 (PMC5829697; doi:10.1590/1677-5449.000417)
Supplement: Translation

# Translation: Oral angioleiomyoma: case report and a review of current findings

**Authors:** Luiz Arthur Barbosa da Silva, Ana Miryam Costa de Medeiros, Patrícia Teixeira de Oliveira, Éricka Janine Dantas da Silveira, Márcia Cristina da Costa Miguel

**Affiliations:** 1 Universidade Federal do Rio Grande do Norte – UFRN, Programa de Pós-graduação em Patologia Oral, Natal, RN, Brazil.

**Keywords:** angioleiomyoma, diagnoses, immunohistochemistry

## Abstract

Angioleiomyoma is a benign neoplasm that was considered a tumor of smooth-muscle origin until the most recent (2013) WHO classification of soft tissue tumors, in which it was reclassified as a tumor of perivascular origin. Angioleiomyomas rarely occur in the oral cavity. These lesions are treated surgically with good prognosis. This article presents a review of reports of oral angioleiomyoma in the literature from the last 5 years and describes the case of a 44-year-old man who presented with an asymptomatic nodule in the upper lip that had developed over a 6-month period. Diagnostic hypotheses of pleomorphic adenoma or canalicular adenoma were raised. Biopsy of the lesion, histopathological and immunohistochemical analysis (S100, CD34, H-caldesmon, and desmin) confirmed a diagnosis of angioleiomyoma. It is noteworthy that immunohistochemistry is an important auxiliary method for differential diagnosis of angioleiomyoma from other tumors, particularly myopericytoma.

## INTRODUCTION

Angioleiomyoma is a benign neoplasm that had been considered a tumor of smooth-muscle origin until the most recent (2013) World Health Organization (WHO) classification of soft tissue tumors, when it was reclassified as a tumor of perivascular origin.^1^ Its etiology remains uncertain, but hypotheses involving minor traumas, venous stasis, hormone dysfunctions, and genetic alterations have been raised.^2^^,^ 3

Angioleiomyomas of the oral cavity are rare, the lips are most frequent site, followed by the palate, mucosa of the cheek, and tongue.^4^^-^ 6 Generally, patients diagnosed with angioleiomyoma are middle-aged adults and it has a preference for males.^4^^,^ 7 ^,^ 8

Clinically, oral angioleiomyoma is characterized as a submucosal nodule, with a firm consistency, slow growth and, in the majority of cases, a size of 2 cm in diameter or less.^8^^,^ 9

Microscopically, it is a well-delimited lesion, with vascular spaces of different sizes and shapes and smooth muscle cells with varying morphology, arranged in disorganized bundles interspersed with collagen fibers.^10^ Differential diagnosis for angioleiomyoma should focus on ruling out myofibroma, neurofibroma, neurilemmoma, leiomyosarcoma and, primarily, myopericytoma. Morphological findings are useful for differentiation when considered together with immunohistochemical results.^5^^,^ 11 ^,^ 12 The most indicated treatment is conservative surgical excision.^6^^,^ 13

The aim of this study was to describe the clinical, morphological, and immunohistochemical findings of a case of oral angioleiomyoma and compare them with findings described in case reports published during the last 5 years in the specialized scientific literature, identified using the PubMed database.

## CASE DESCRIPTION

A male, 44-year-old, melanoderm patient sought care at an oral diagnosis service, presenting an asymptomatic increase in volume, with a lobulated surface, normochromic mucosal, fibrous consistency, located on the right side of the upper lip, and with duration of approximately 6 months (Figure 1). His medical and family histories were not relevant to the case. After clinical examination, the diagnostic hypotheses were canalicular adenoma and pleomorphic adenoma. Under local anesthesia, an excisional biopsy of the lesion was conducted with no complications. The specimen removed was fixed in 10% formol and sent to a pathology laboratory. Examination of histological sections revealed a benign neoplasm of mesenchymal origin, located in a subepithelial region and characterized by proliferation of cells with a variety of morphologies (ovoid, fusiform, and ondulated), arranged in disorganized bundles that surrounded multiple blood vessels of several calibers (Figure 2A). The clinical hypotheses of benign salivary gland neoplasms (adenoma pleomorphic or canalicular adenoma) were rejected. Immunohistochemical analyses were conducted to confirm the diagnosis (Table 1). The tumor cells were negative for S100 (Figure 2B), and positive staining for CD34 was restricted to the walls of blood vessels (Figure 2C). The tumor cells had intense staining for α-SMA (Figure 2D), and were immunopositive for H-caldesmon (Figure 2E) and desmin (Figure 2F). A diagnosis of oral angioleiomyoma was confirmed based on these findings. Fifteen months later the patient is still being followed-up clinically and there have been no signs of relapse.

**Figure 1:** Clinical appearance: nodular lesion on upper lip.

**Figure 2:** Morphological and immunohistochemical features of angioleiomyoma. (A) proliferation of cells with varied morphology (ovoid, fusiform, and ondulated) arranged in disorganized bundles and surrounding multiple blood vessels (Hematoxylin & eosin, 100 µm); (B) Absence of immunostaining of tumor cells for S100 protein; (C) Positive staining for CD34 restricted to vascular walls; (D-F) Strong and diffuse immunostaining for proteins α-SMA, H-caldesmon, and desmin, respectively (100 µm).

**Table 1:** Immunohistochemical panel.

Antibody	Manufacturer	Dilution and incubation	Findings
S100	Dako	1:2000	Negative for tumor cells
		60 minutes	
CD34	Dako	1:200	Restricted to vessel walls
		60 minutes	
α-SMA	Dako	1:300	Strong and diffuse
		Overnight	
H-caldesmon	Dako	1:400	Strong and diffuse
		60 minutes	
Desmin	Dako	1:400	Strong and diffuse
		60 minutes	

## DISCUSSION

According to the WHO, angioleiomyomas are benign dermal or subcutaneous tumors made up of well-differentiated smooth muscle cells that organize around a variety of vascular structures.^14^ Their etiology remains to be confirmed. Although genetic studies related to the origin of perivascular lesions are still limited, mutations to the BRAF, NF1, NOTCH2 and NOTCH3 genes are under investigation.^1^ Additionally, estrogen receptor and progesterone receptor expression have been investigated with relation to angioleiomyomas, on the basis that there is a possibility that hormonal changes participate as an etiologic factor.^2^

Angioleiomyomas can arise on any part of the body, but in the majority of cases they occur in extremities, primarily the lower limbs, followed by the head and trunk.^8^ High incidences are also reported in the uterus, gastrointestinal tract, and skin.^4^^,^ 6 Angioleiomyomas are rare in the oral cavity, since smooth muscle is scarce in this region.^9^ These tumors appear to arise from the tunica media of small vessels, from arteriovenous anastomoses, or the smooth muscle cells of the circumvallate papillae of the tongue.^5^^,^ 12 Although the lips are described as the most common site of angioleiomyomas within the oral cavity,^4^^-^ 6 according to reports in the specialized literature published over the last 5 years (summarized in Table 2), the gingiva was the most common anatomical site in 29.4% of cases.

**Table 2:** Reports of oral angioleiomyoma cases reported in the literature over the last 5 years (2011-2016).

Authors	N	Sex	Age (Years)	Anatomic site	Symptomology
Present study (2016)	1	Male	44	Lower lip	Asymptomatic
Arpağ et al.^5^	2	Male	25	Gingiva	Asymptomatic
		Female	55	Gingiva	
Bajpai et al.^13^	1	Male	39	Gingiva	Asymptomatic
Ishikawa et al.^6^	1	Male	51	Tongue	Asymptomatic
Inaba et al.^2^	1	Female	45	Mucosa of cheek	Asymptomatic
Osano et al.^3^	1	Male	45	Mucosa of cheek	Asymptomatic
Ranjan and Singh^9^	1	Female	45	Gingiva	Asymptomatic
Tsuji et al.^4^	1	Male	79	Hard palate	Asymptomatic
Eley et al.^8^	1	Male	39	Hard palate	Asymptomatic
Menditti et al.^15^	1	Male	14	Gingiva	Asymptomatic
Gueiros et al.^10^	3	Male	53	Upper lip	Asymptomatic
		Male	54	Lower lip	Asymptomatic
		Male	66	Upper lip	Asymptomatic
Mahima et al.^12^	1	Male	57	Retromolar area	Asymptomatic
Reddy et al.^7^	1	Male	9	Mandible	Asymptomatic
Vidaković et al.^16^	1	Female	85	Parotid	Asymptomatic

N: number of cases.

Oral angioleiomyoma is more common in male patients,^4^^,^ 8 with a male:female ratio of 3:1.^3^ Normally, the peak incidence of this tumor is between the 4th and 6th decades of life;^8^^,^ 10 but there are cases in pediatric patients.^7^ In the review summarized in Table 2, it was observed that 76.4% of the tumors were in males, and patients’ ages ranged from 9 to 85 years, with a mean age of 47.3 years. The patient in the present study was a 44-year-old male.

The clinical findings in our case corroborate those described in previous literature. Angioleiomyomas present as asymptomatic submucosal nodules with slow growth, that are well-defined, mobile, occasionally with a bluish color and intact surface, and generally measure 2 cm in diameter.^7^^,^ 9

The immunohistochemical profile described here confirms the muscular origin of the neoplastic cells in this tumor, since strong and diffuse immunostaining was observed for myogenic proteins, such as α-SMA, H-caldesmon, and desmin. The most important differential diagnosis for angioleiomyoma is myopericytoma, because these two types of lesion have overlapping histopathological findings.^9^ In one study evaluating 122 cases of angioleiomyoma and 12 cases of myopericytoma, Matsuyama, Hisaoka and Hashimoto^11^ attempted to determine the characteristic immunohistochemical profiles of these two tumors, observing that α-SMA, HHF-35, and H-caldesmon exhibited the same immunostaining profile, whereas desmin was negative in 75% of myopericytoma cases and just 17.1% of angioleiomyoma cases. Therefore, desmin appears to be a useful marker for differentiation between these two lesions. From an immunohistochemical point of view, desmin may also be useful for differentiating angioleiomyomas from myofibromas, since the neoplastic cells in myofibromas are negative for this marker.^9^

The profile of immunostaining for the S100 protein is also useful for differentiating a diagnosis of angioleiomyoma from neoplasms such as neurofibroma and neurilemmoma, since in addition to the morphological profile of these lesions, the fact that the tumor cells of angioleiomyomas fail to react to this protein is entirely divergent from what is observed with lesions of neural origin.^15^

Histopathological differentiation between angioleiomyoma and low grade leiomyosarcoma may be difficult. Among other parameters, leiomyosarcomas can be identified by mitosis counts ranging from 5 to 10 per field, cell nuclei with blunt ends, and foci of necrosis. Therefore, careful long-term follow-up is necessary in view of the diagnostic uncertainty between these two entities.^3^^,^ 15

The treatment of choice for oral angioleiomyoma is conservative surgical excision. Rare recurrence has been described, probably the result of incomplete surgical excision. There are no reports of malignant transformation and patients’ prognosis is considered excellent.^6^^,^ 16 The patient described here has been in follow-up for 15 months with no evidence of relapse.

Awareness of the WHO^14^ reclassification of angioleiomyoma as a tumor of perivascular origin is important and more genetic studies are needed to elucidate their true etiology. Immunohistochemical studies are an important method to aid in arriving at the correct diagnosis of angioleiomyoma, particularly with respect to differential diagnosis from other lesions, especially myopericytoma.
